# Supplementary material for: LRP5, SLC6A3, and SOX10 Expression in Conventional Ameloblastoma
Source: Genes (Basel). 2023 Jul 26;14(8):1524. doi: 10.3390/genes14081524 (PMC10453908; doi:10.3390/genes14081524)
Supplement: Supplementary file 1 [file genes-14-01524-s001.zip › genes-2491286-supplementary.pdf]

Supplementary table 1. List of P value and Log Fold Changes of ameloblastoma profile genes		
**Symbol**	**PValue**	**logFC**
OBP2A	2.36E-18	-2.9893191
LGALS4	2.92E-18	-2.9347697
NT5M	5.66E-19	-2.8690479
LILRB1	9.69E-18	-2.7996894
FLJ20343	1.03E-17	-2.7255629
DNAH9	5.93E-18	-2.7078208
EIF4ENIF1	2.62E-18	-2.6816422
IFNA8	3.41E-18	-2.6701346
FLJ10587	5.43E-18	-2.6563194
ASRGL1	3.50E-18	-2.5959203
ATP5H	1.72E-17	-2.5653845
PIGL	1.07E-17	-2.5191319
CBLC	1.27E-17	-2.489802
MX2	1.92E-17	-2.4737391
JIK	1.02E-17	-2.4533314
EPN3	2.97E-18	-2.4532638
SRP14	4.86E-17	-2.4521452
HIST1H1E	1.53E-17	-2.4358077
RAI	1.42E-17	-2.4047196
FLJ20530	1.33E-17	-2.395883
RPL29	9.56E-18	-2.3938102
HLF	1.38E-16	-2.3855171
MICA	1.18E-17	-2.3849491

SUCLA2	1.91E-17	-2.3845861
PHF11	3.99E-17	-2.3675199
MRPL39	1.56E-16	-2.362284
SEC10L1	2.20E-18	-2.3483933
FLJ20045	2.40E-17	-2.3292114
PTK9	1.73E-17	-2.3249519
NRG1	6.94E-18	-2.3076019
PTPRO	8.12E-18	-2.2907231
CTSB	6.03E-17	-2.2802467
PHF3	1.45E-17	-2.2793134
FABP5	2.60E-17	-2.2790737
DUSP3	9.70E-18	-2.2738341
PSTPIP2	1.69E-17	-2.2686714
TADA2L	7.34E-17	-2.2545662
HPS5	8.56E-18	-2.2482819
RPL37	3.39E-17	-2.1453696
DNMT3A	1.73E-17	-2.1227859
TMEM14B	3.95E-18	-2.0924561
CAS1	1.83E-16	-2.0695977
SYT5	3.89E-17	-2.064543
MC4R	1.62E-17	-2.0637994
YWHAZ	2.67E-17	-2.0461605
SLC14A1	6.06E-17	-2.0459744
GLE1L	6.33E-16	-2.0364516
SUPT16H	5.62E-17	-2.0257353
BTN2A2	4.11E-16	-2.0254121
ZNF180	2.94E-16	-2.0216184

SAP18	6.04E-16	2.00329808
SFXN1	5.52E-17	2.00574822
CEPT1	1.45E-15	2.01052054
CLCN6	4.01E-17	2.01256685
ZNF195	1.54E-16	2.02032268
AK2	6.01E-17	2.02379827
MGC3169	2.61E-17	2.03019288
NTSR1	9.54E-17	2.0404398
PSMB2	1.49E-16	2.04344903
TAS2R13	1.17E-16	2.04834847
PPHLN1	5.37E-17	2.05241771
RCP	8.83E-17	2.05429376
HRPT2	8.32E-17	2.05530649
B3GAT3	1.35E-16	2.06038305
KRTHA7	6.90E-17	2.06320633
MTA2	8.55E-17	2.06546133
MYO5C	3.18E-16	2.07345118
STK25	1.02E-16	2.0753425
CTAGE1	5.51E-16	2.07634974
C7	1.98E-17	2.07707622
GTF3C2	3.43E-17	2.08051663
TAS2R1	4.54E-17	2.09188739
UPF3A	3.77E-17	2.09271133
QPCT	2.11E-17	2.09502399
DKKL1	1.57E-16	2.09879532
DRD3	5.50E-17	2.10028558
ELF4	6.78E-17	2.1070931

KCNJ4	4.50E-16	2.10780242
PDZK2	5.97E-17	2.11016307
USP36	3.03E-17	2.11017679
WDR4	4.05E-17	2.110357
PLXNC1	8.64E-17	2.11438653
COP1	2.24E-16	2.11468418
FLRT1	6.16E-17	2.12137752
HMG20B	5.31E-16	2.12140287
FGFR1	2.90E-17	2.13010528
MLLT1	3.90E-17	2.13327875
FLJ20519	3.47E-17	2.14144062
TREX2	6.52E-17	2.15015886
SF3A1	1.30E-17	2.15124917
RANBP3	1.56E-17	2.1554372
BPI	8.72E-18	2.15679823
Bit1	6.98E-16	2.16314246
DSG3	3.16E-16	2.17504474
GP1BA	1.23E-17	2.1772155
AAK1	2.15E-17	2.17968305
PLA2G5	1.06E-16	2.18216005
ARHGDIA	4.35E-16	2.18290977
FLJ10874	8.42E-18	2.18782214
GRIN2D	1.19E-16	2.19029009
HNF4G	8.54E-17	2.19412655
SF3B4	8.85E-18	2.19591995
HNRPH3	4.82E-17	2.1989628
IRAK2	5.49E-17	2.20266286

LILRB3	3.78E-16	2.21368532
CRYBB1	9.47E-18	2.22003865
SLC22A8	5.68E-17	2.22295982
RNF110	1.62E-16	2.22509986
LGALS3BP	6.50E-17	2.22524507
ITGBL1	3.17E-17	2.22706588
PLD2	4.81E-17	2.22730753
ITGB4BP	1.02E-16	2.24674944
CDY	1.90E-17	2.24775691
NBL1	7.79E-17	2.2534961
FLJ11164	4.12E-17	2.25731766
IKBKG	1.64E-16	2.26140621
ECGF1	1.22E-17	2.26372536
NKX2-8	8.30E-18	2.26810384
ADMR	8.37E-16	2.2704886
SDCBP2	8.32E-17	2.2794525
FLJ10761	6.79E-17	2.28358939
CGI-85	5.30E-17	2.2937176
FLJ20331	1.52E-16	2.29574449
MART2	1.89E-17	2.30513643
FLJ10305	2.39E-17	2.30918829
CDC20	2.08E-17	2.31728696
PBX3	7.79E-17	2.33355605
SOX8	2.64E-17	2.3335974
CCDC6	4.47E-18	2.34715435
SPIN2	1.32E-17	2.34884572
FA2H	3.46E-17	2.35256617

MAOB	1.60E-17	2.35806573
LRP5	3.96E-17	2.36246394
PIGN	9.88E-17	2.36982252
CHST6	3.69E-19	2.3712318
HSRG1	3.77E-18	2.37987054
ATP5G1	8.63E-18	2.39460089
HBD	7.94E-16	2.39876301
GABRD	2.49E-17	2.40300176
NR4A1	1.46E-17	2.40874381
FLJ20359	2.08E-17	2.42151779
PEF	4.52E-17	2.42572343
SYNGR1	2.08E-16	2.43053541
FPRL1	1.58E-17	2.43226863
SMARCA3	4.84E-16	2.44454187
TCAP	2.84E-17	2.44922815
OPTC	2.47E-17	2.46184292
GATM	2.38E-16	2.46431929
TLX3	2.72E-17	2.47027566
DKFZP566B183	1.73E-17	2.4771584
GPR44	1.44E-16	2.47725496
MYO5A	7.60E-17	2.47768658
ZNF426	9.16E-17	2.4790135
GRK6	1.84E-17	2.48850782
GYS1	2.16E-17	2.49107765
DPF2	3.85E-17	2.49343171
FBXL8	2.97E-17	2.49740084
ZCWCC2	2.50E-17	2.49991591

SYN1	8.93E-18	2.5064294
TIMM17A	9.38E-17	2.5066203
JARID1B	4.60E-17	2.50929963
WDR8	2.90E-16	2.51059502
MC1R	3.50E-17	2.5289621
MAGEB4	2.10E-17	2.55214565
MAP1A	9.06E-18	2.58212247
FEZ1	1.07E-17	2.60210037
CRHR2	9.03E-19	2.60253496
DIO3	4.84E-17	2.60619543
HS3ST3A1	1.74E-17	2.6108714
E2F4	5.65E-18	2.62300577
GORASP2	1.74E-17	2.6345349
FLJ11856	5.95E-18	2.63524616
KIAA0040	9.92E-18	2.639522
MAN2A2	1.87E-16	2.65355333
PAK1	2.20E-17	2.66211713
FOS	2.36E-17	2.66648467
KRTHA2	3.32E-17	2.67514935
XCR1	7.59E-17	2.68312598
GDF1	4.80E-18	2.68956973
TNFSF7	1.70E-17	2.69378577
GARP	3.75E-18	2.70260486
ARAP3	3.93E-18	2.71335259
GPR135	1.18E-16	2.7168394
PYCR1	4.28E-18	2.72371626
PDHA1	2.24E-17	2.72599082

KRTHA1	1.56E-16	2.73587651
FOXK2	3.40E-16	2.75202094
CTAG1B	1.12E-17	2.75339909
UBQLN3	1.03E-17	2.75681438
CDH1	4.53E-19	2.78915411
KLF8	8.94E-18	2.79814367
TOP2B	5.78E-18	2.81616414
CLDN12	7.94E-18	2.82692979
PHF1	3.53E-17	2.8290569
SOX10	2.92E-18	2.85855242
GMEB2	6.97E-17	2.86764371
ALG3	1.57E-17	2.87291486
MICAL2	9.61E-18	2.89161726
BCKDK	7.98E-17	2.90185755
MFNG	5.09E-17	2.92891435
USP1	2.68E-17	2.96199776
LIPE	7.31E-17	2.96314113
NR4A3	3.19E-17	2.96741497
DAB2	1.56E-18	2.98430045
EFNA3	1.96E-18	2.98458127
CRA	5.77E-18	2.99405993
PPY2	3.11E-18	3.00937552
DVL2	2.56E-18	3.01039684
NAG18	1.39E-18	3.01538105
EIF3S4	3.13E-18	3.04396438
pp9099	3.90E-18	3.05822593
POU5F1	6.80E-18	3.07652475

DLL3	7.53E-18	3.10711227
FLII	6.67E-17	3.13931391
RNF5	2.15E-18	3.14182047
GREM1	4.05E-18	3.15924058
IL17R	5.02E-17	3.1688108
GPR56	4.37E-17	3.18687796
ARSA	7.43E-17	3.20345866
FLJ11286	3.30E-18	3.21216492
ABHD4	1.24E-17	3.21665694
MGC3047	3.95E-18	3.23020528
STAT1	8.81E-18	3.23085491
GNRH2	5.03E-18	3.24451131
EFNA4	7.67E-18	3.26129129
MCOLN1	6.49E-17	3.26279844
ADRA1D	2.46E-18	3.26781575
SERF1A	2.03E-17	3.28629473
ZNFN1A5	1.95E-18	3.31234549
AQP5	7.30E-18	3.33982686
NDP	2.25E-18	3.3410905
HOXD4	1.28E-18	3.3584878
SIAT8E	9.65E-18	3.36930581
APOM	5.08E-18	3.3699619
OAS1	8.75E-19	3.40979339
FXYD3	4.07E-18	3.45265673
KRTHA5	1.08E-17	3.46552511
PRSS8	9.11E-18	3.50206126
FLJ20249	1.83E-17	3.51299099

GPS2	9.22E-18	3.60133074
SSTR3	4.21E-18	3.6416582
TESK1	2.19E-18	3.66237453
KIF3B	2.28E-18	3.69072198
EDG5	4.31E-18	3.72560629
UTF1	7.90E-20	3.73044959
GPR3	1.35E-18	3.7426546
ARHGAP8	3.28E-18	3.74287319
ENTPD1	1.29E-18	3.90427326
SLC6A3	5.49E-19	3.94245919
DLGAP1	7.02E-18	4.09086984
AP1G2	4.17E-18	4.21903218
NKG7	9.20E-19	4.37589591

Supplementary table 2. Enrichment criteria of conventional ameloblastoma

Enrichment FDR	**n Genes**	**Pathway Genes**	**Fold Enrichment**	**Pathway**	**URL**	**Genes**
**0.00132964**	30	1264	2.77458617	Positive regulation of transcription by RNA polymerase II	http://amigo.geneontology.org/amigo/term/GO:0045944	DVL2 SOX8 SUPT16H SOX10 GMEB2 ELF4 HLF STAT1 NR4A3 NR4A1 MICAL2 DPF2 NKX2-8 FOXK2 MTA2 DRD3 DAB2 LRP5 HNF4G PBX3 HOXD4 FOS UTF1 CTAG1B E2F4 POU5F1 MC1R IKBKG GREM1 GPS2
**0.01416068**	3	6	58.4512821	Response to corticotropin-releasing hormone	http://amigo.geneontology.org/amigo/term/GO:0043435	CRHR2 NR4A3 NR4A1
**0.01416068**	3	6	58.4512821	Cellular response to corticotropin-releasing hormone stimulus	http://amigo.geneontology.org/amigo/term/GO:0071376	CRHR2 NR4A3 NR4A1
**0.01423179**	32	1716	2.1800012	Positive regulation of transcription, DNA-templated	http://amigo.geneontology.org/amigo/term/GO:0045893	DVL2 SOX8 CDH1 SUPT16H SOX10 GMEB2 ELF4 HLF STAT1 NR4A3 NR4A1 NDP MICAL2 DPF2 NKX2-8 FOXK2 MTA2 DRD3 DAB2 LRP5 HNF4G PBX3

						HOXD4 FOS UTF1 CTAG1B E2F4 POU5F1 MC1R IKBKG GREM1 GPS2
**0.01423179**	32	1717	2.17873154	Positive regulation of RNA biosynthetic process	http://amigo.geneontology.org/amigo/term/GO:1902680	DVL2 SOX8 CDH1 SUPT16H SOX10 GMEB2 ELF4 HLF STAT1 NR4A3 NR4A1 NDP MICAL2 DPF2 NKX2-8 FOXK2 MTA2 DRD3 DAB2 LRP5 HNF4G PBX3 HOXD4 FOS UTF1 CTAG1B E2F4 POU5F1 MC1R IKBKG GREM1 GPS2
**0.01423179**	32	1716	2.1800012	Positive regulation of nucleic acid-templated transcription	http://amigo.geneontology.org/amigo/term/GO:1903508	DVL2 SOX8 CDH1 SUPT16H SOX10 GMEB2 ELF4 HLF STAT1 NR4A3 NR4A1 NDP MICAL2 DPF2 NKX2-8 FOXK2 MTA2 DRD3 DAB2 LRP5 HNF4G PBX3 HOXD4 FOS UTF1 CTAG1B E2F4 POU5F1 MC1R IKBKG GREM1 GPS2
**0.01429234**	34	1895	2.09746025	Positive regulation of RNA metabolic process	http://amigo.geneontology.org/amigo/term/GO:0051254	DVL2 SOX8 CDH1 SUPT16H SOX10 GMEB2 ELF4 HLF STAT1 NR4A3 NR4A1 NDP MICAL2 DPF2 NKX2-8 FOXK2 SF3B4 MTA2 DRD3 DAB2 LRP5 HNF4G PBX3 HOXD4 FOS UTF1 CTAG1B EIF4ENIF1 E2F4 POU5F1 MC1R IKBKG GREM1 GPS2
**0.0167191**	32	1757	2.12913037	Neurogenesis	http://amigo.geneontology.org/amigo/term/GO:0022008	DVL2 SOX8 SYN1 CDH1 HMG20B TOP2B FGFR1 DLL3 SOX10 FA2H STK25 CDC20 DNMT3A PLXNC1 NKX2-8 ARHGDIA EFNA3 PAK1 FEZ1 PTPRO DRD3 NRG1 NBL1 TLX3 YWHAZ MAP1A PBX3 FPR2 EIF4ENIF1 DIO3 ADGRG1 EFNA4
**0.02074168**	30	1624	2.15953013	Generation of neurons	http://amigo.geneontology.org/amigo/term/GO:0048699	DVL2 SOX8 SYN1 CDH1 HMG20B TOP2B FGFR1 DLL3 SOX10 STK25 CDC20 DNMT3A PLXNC1 NKX2-8 ARHGDIA EFNA3 PAK1 FEZ1 PTPRO DRD3 NRG1 NBL1 TLX3 YWHAZ MAP1A PBX3 EIF4ENIF1 DIO3 ADGRG1 EFNA4
**0.0457034**	32	1885	1.98455281	Cell-cell signaling	http://amigo.geneontology.org/amigo/term/GO:0007267	DVL2 SYN1 CDH1 MAOB SOX10 SYNGR1 NTSR1 GRIN2D CRHR2 CDC20 NDP PSMB2 SYT5 EFNA3 PTPRO DRD3 DAB2 NRG1 LRP5 FABP5 MAP1A DLGAP1 ADRA1D GABRD GRK6 ADGRG1 POU5F1 EFNA4 S1PR2 GREM1 SLC6A3 SSTR3
**0.0457034**	33	1970	1.9582663	Positive regulation of macromolecule biosynthetic process	http://amigo.geneontology.org/amigo/term/GO:0010557	DVL2 SOX8 CDH1 SUPT16H SOX10 GMEB2 ELF4 HLF STAT1 NR4A3 NR4A1 NDP MICAL2 DPF2 NKX2-8 FOXK2 MTA2 DRD3 DAB2 LRP5 HNF4G PBX3 UPF3A HOXD4 FOS UTF1 CTAG1B E2F4 POU5F1 MC1R IKBKG GREM1 GPS2
**0.05149687**	13	460	3.30376812	Pattern specification process	http://amigo.geneontology.org/amigo/term/GO:0007389	DVL2 DLL3 MFNG KIF3B MICAL2 NBL1 LRP5 PBX3 HOXD4 TCAP ADGRG1 POU5F1 GREM1
**0.05823235**	9	243	4.3297246	Adenylate cyclase-modulating G protein-coupled	http://amigo.geneontology.org/amigo/term/GO:0007188	CRHR2 DRD3 MC4R FPR2 ADRA1D GPR3 ADGRG1 MC1R S1PR2

				receptor signaling pathway		
**0.05823235**	15	606	2.89362782	Regulation of system process	http://amigo.geneontology.org/amigo/term/GO:0044057	FXYD3 SOX10 NTSR1 GRIN2D NR4A3 PAK1 PTPRO DRD3 TLX3 FABP5 PBX3 KCNJ4 DLGAP1 ADRA1D S1PR2
**0.05823235**	19	897	2.47619701	Positive regulation of cell differentiation	http://amigo.geneontology.org/amigo/term/GO:0045597	SOX8 HMG20B FGFR1 SOX10 TESK1 STAT1 STK25 PLA2G5 PLXNC1 ARHGDIA PAK1 FEZ1 DAB2 NRG1 NBL1 LRP5 FOS S1PR2 GREM1
**0.05823235**	2	3	77.9350427	Norrin signaling pathway	http://amigo.geneontology.org/amigo/term/GO:0110135	NDP LRP5
**0.05919308**	7	149	5.49206677	Adenylate cyclase-activating G protein-coupled receptor signaling pathway	http://amigo.geneontology.org/amigo/term/GO:0007189	DRD3 MC4R ADRA1D GPR3 ADGRG1 MC1R S1PR2
**0.05919308**	5	68	8.59577677	Skeletal muscle cell differentiation	http://amigo.geneontology.org/amigo/term/GO:0035914	SOX8 HMG20B HLF NR4A1 FOS
**0.06600716**	26	1473	2.06345327	Neuron differentiation	http://amigo.geneontology.org/amigo/term/GO:0030182	DVL2 SOX8 SYN1 CDH1 HMG20B TOP2B FGFR1 STK25 CDC20 DNMT3A PLXNC1 NKX2-8 ARHGDIA EFNA3 PAK1 FEZ1 PTPRO NRG1 NBL1 TLX3 YWHAZ MAP1A PBX3 EIF4ENIF1 DIO3 EFNA4
**0.07222863**	2	4	58.4512821	Compartment pattern specification	http://amigo.geneontology.org/amigo/term/GO:0007386	DLL3 PBX3
**0.07222863**	2	4	58.4512821	Golgi reassembly	http://amigo.geneontology.org/amigo/term/GO:0090168	STK25 YWHAZ
**0.11965066**	29	1982	1.82844576	Locomotion	http://amigo.geneontology.org/amigo/term/GO:0040011	SOX8 CDH1 TOP2B FGFR1 SOX10 TESK1 DUSP3 NR4A3 NR4A1 PLD2 PLXNC1 EFNA3 PAK1 MTA2 FEZ1 PTPRO DRD3 PSTPIP2 DAB2 NBL1 LRP5 TLX3 FPR2 XCR1 ITGBL1 ADGRG1 EFNA4 S1PR2 GREM1
**0.12320477**	3	22	15.9412587	Postsynaptic neurotransmitter receptor internalization	http://amigo.geneontology.org/amigo/term/GO:0098884	DRD3 NRG1 MX2
**0.12320477**	3	22	15.9412587	Postsynaptic endocytosis	http://amigo.geneontology.org/amigo/term/GO:0140239	DRD3 NRG1 MX2
**0.12433392**	28	1738	1.88335546	Regulation of cell differentiation	http://amigo.geneontology.org/amigo/term/GO:0045595	SOX8 HMG20B FGFR1 DLL3 SOX10 TESK1 STAT1 STK25 NR4A3 PSMB2 PLA2G5 DPF2 PLXNC1 ARHGDIA PAK1 FEZ1 DRD3 DAB2 NRG1 NBL1 LRP5 TLX3 FOS EIF4ENIF1 GP1BA S1PR2 GREM1 GPS2

**0.12515295**	10	352	3.32109557	Regionalization	http://amigo.geneontology.org/amigo/term/GO:0003002	DVL2 DLL3 NBL1 LRP5 PBX3 HOXD4 TCAP ADGRG1 POU5F1 GREM1
**0.12515295**	3	23	15.2481605	Regulation of respiratory gaseous exchange	http://amigo.geneontology.org/amigo/term/GO:0043576	NTSR1 TLX3 PBX3
**0.13878656**	24	1590	1.91161426	Cell migration	http://amigo.geneontology.org/amigo/term/GO:0016477	SOX8 CDH1 TOP2B FGFR1 SOX10 TESK1 DUSP3 NR4A3 NR4A1 PLXNC1 PAK1 MTA2 PTPRO PSTPIP2 DAB2 NBL1 LRP5 TLX3 FPR2 XCR1 ITGBL1 ADGRG1 S1PR2 GREM1
**0.15727961**	3	27	12.9891738	DNA methylation-dependent heterochromatin assembly	http://amigo.geneontology.org/amigo/term/GO:0006346	DNMT3A PPHLN1 POU5F1
**0.15727961**	6	149	4.7074858	Regulation of nervous system process	http://amigo.geneontology.org/amigo/term/GO:0031644	SOX10 NTSR1 GRIN2D FABP5 DLGAP1 S1PR2
**0.15727961**	3	26	13.4887574	Regulation of osteoblast proliferation	http://amigo.geneontology.org/amigo/term/GO:0033688	SOX8 LRP5 GREM1
**0.15727961**	3	26	13.4887574	Cell death in response to hydrogen peroxide	http://amigo.geneontology.org/amigo/term/GO:0036474	STK25 NR4A3 PYCR1
**0.15727961**	2	8	29.225641	D-amino acid transport	http://amigo.geneontology.org/amigo/term/GO:0042940	NTSR1 SFXN1
**0.15727961**	7	197	4.15389822	Regulation of neuron differentiation	http://amigo.geneontology.org/amigo/term/GO:0045664	SOX8 HMG20B FGFR1 FEZ1 NBL1 TLX3 EIF4ENIF1
**0.15727961**	5	102	5.73051785	Oligodendrocyte differentiation	http://amigo.geneontology.org/amigo/term/GO:0048709	SOX8 SOX10 FA2H DRD3 NRG1
**0.15727961**	23	1373	1.95830952	Positive regulation of developmental process	http://amigo.geneontology.org/amigo/term/GO:0051094	DVL2 SOX8 HMG20B FGFR1 SOX10 TESK1 STAT1 STK25 CDC20 PLA2G5 PLXNC1 ARHGDIA PAK1 FEZ1 DAB2 NRG1 NBL1 LRP5 FOS DIO3 POU5F1 S1PR2 GREM1
**0.15727961**	9	324	3.24729345	Negative regulation of cellular component movement	http://amigo.geneontology.org/amigo/term/GO:0051271	CDH1 DUSP3 ARHGDIA PTPRO NRG1 NBL1 ADGRG1 S1PR2 GREM1
**0.15727961**	2	8	29.225641	Establishment of Golgi localization	http://amigo.geneontology.org/amigo/term/GO:0051683	STK25 YWHAZ

**0.15727961**	2	8	29.225641	Negative regulation of monocyte chemotaxis	http://amigo.geneontology.org/amigo/term/GO:0090027	NBL1 GREM1
**0.15727961**	16	815	2.29501966	Synaptic signaling	http://amigo.geneontology.org/amigo/term/GO:0099536	SYN1 CDH1 SYNGR1 NTSR1 GRIN2D CRHR2 CDC20 SYT5 DRD3 NRG1 FABP5 MAP1A DLGAP1 GABRD S1PR2 SLC6A3
**0.15727961**	3	29	12.0933687	Neurotransmitter receptor internalization	http://amigo.geneontology.org/amigo/term/GO:0099590	DRD3 NRG1 MX2
**0.15727961**	2	8	29.225641	Regulation of peptidyl-tyrosine autophosphorylation	http://amigo.geneontology.org/amigo/term/GO:1900084	NRG1 GREM1
**0.15727961**	2	7	33.4007326	Positive regulation of peptidyl-tyrosine autophosphorylation	http://amigo.geneontology.org/amigo/term/GO:1900086	NRG1 GREM1
**0.15727961**	2	8	29.225641	Peptidyl-tyrosine dephosphorylation involved in inactivation of protein kinase activity	http://amigo.geneontology.org/amigo/term/GO:1990264	DUSP3 PTPRO
**0.18125483**	3	31	11.3131514	Cell proliferation	http://amigo.geneontology.org/amigo/term/GO:0033687	SOX8 LRP5 GREM1
**0.1819118**	2	9	25.9783476	Positive regulation of peroxisome proliferator activated receptor signaling pathway	http://amigo.geneontology.org/amigo/term/GO:0035360	FABP5 GPS2
**0.1819118**	26	1776	1.77723493	Cell motility	http://amigo.geneontology.org/amigo/term/GO:0048870	SOX8 CDH1 TOP2B FGFR1 SOX10 TESK1 DUSP3 NR4A3 NR4A1 PLD2 PLXNC1 PAK1 MTA2 PTPRO PSTPIP2 DAB2 NBL1 LRP5 TLX3 FPR2 XCR1 ITGBL1 ADGRG1 S1PR2 GREM1 SLC6A3
**0.1819118**	27	1776	1.77723493	Localization of cell	http://amigo.geneontology.org/amigo/term/GO:0051674	SOX8 CDH1 TOP2B FGFR1 SOX10 TESK1 DUSP3 NR4A3 NR4A1 PLD2 PLXNC1 ARHGDIA PAK1 MTA2 PTPRO PSTPIP2 DAB2 NRG1 NBL1 LRP5 TLX3 FPR2 XCR1 ITGBL1 ADGRG1 S1PR2 GREM1
**0.1819118**	11	471	2.7302085	Regulation of trans-synaptic signaling	http://amigo.geneontology.org/amigo/term/GO:0099177	SYN1 CDH1 SYNGR1 GRIN2D CRHR2 CDC20 DRD3 FABP5 MAP1A DLGAP1 S1PR2

**0.19293439**	5	114	5.12730544	Phenol-containing compound metabolic process	http://amigo.geneontology.org/amigo/term/GO:0018958	MAOB DRD3 CTSB DIO3 MC1R
**0.19293439**	2	10	23.3805128	Sequestering of BMP from receptor via BMP binding	http://amigo.geneontology.org/amigo/term/GO:0038098	NBL1 GREM1
**0.19293439**	5	115	5.08272018	Regulation of receptor-mediated endocytosis	http://amigo.geneontology.org/amigo/term/GO:0048259	AAK1 DRD3 DAB2 NRG1 GREM1
**0.19293439**	2	10	23.3805128	Determination of dorsal identity	http://amigo.geneontology.org/amigo/term/GO:0048263	NBL1 GREM1
**0.19293439**	3	34	10.3149321	Arachidonic acid secretion	http://amigo.geneontology.org/amigo/term/GO:0050482	NTSR1 PLA2G5 DRD3
**0.19293439**	4	69	6.77696024	Negative regulation of chemotaxis	http://amigo.geneontology.org/amigo/term/GO:0050922	DUSP3 PTPRO NBL1 GREM1
**0.19293439**	15	776	2.25971451	Trans-synaptic signaling	http://amigo.geneontology.org/amigo/term/GO:0099537	SYN1 CDH1 SYNGR1 NTSR1 GRIN2D CRHR2 CDC20 SYT5 DRD3 FABP5 MAP1A DLGAP1 GABRD S1PR2 SLC6A3
**0.19293439**	3	34	10.3149321	Arachidonate transport	http://amigo.geneontology.org/amigo/term/GO:1903963	NTSR1 PLA2G5 DRD3
**0.19304155**	4	71	6.58605995	Respiratory gaseous exchange by respiratory system	http://amigo.geneontology.org/amigo/term/GO:0007585	NTSR1 TLX3 YWHAZ PBX3
**0.19304155**	9	350	3.00606593	Negative regulation of locomotion	http://amigo.geneontology.org/amigo/term/GO:0040013	CDH1 DUSP3 ARHGDIA PTPRO NRG1 NBL1 ADGRG1 S1PR2 GREM1
**0.19304155**	3	35	10.0202198	Regulation of monocyte chemotaxis	http://amigo.geneontology.org/amigo/term/GO:0090025	NBL1 FPR2 GREM1
**0.19423342**	13	637	2.38576661	Endocytosis	http://amigo.geneontology.org/amigo/term/GO:0006897	EPN3 LGALS3BP AAK1 PLA2G5 SYT5 DRD3 DAB2 NRG1 LRP5 FPR2 MX2 AP1G2 GREM1
**0.19423342**	11	490	2.62434328	Axonogenesis	http://amigo.geneontology.org/amigo/term/GO:0007409	TOP2B STK25 PLXNC1 NKX2-8 ARHGDIA EFNA3 PAK1 FEZ1 PTPRO MAP1A EFNA4
